# The Diagnostic and Prognostic Utility of Contemporary Cardiac Magnetic Resonance in Suspected Acute Myocarditis

**DOI:** 10.3390/diagnostics12010156

**Published:** 2022-01-10

**Authors:** Jakub Lagan, Christien Fortune, David Hutchings, Joshua Bradley, Josephine H. Naish, Richard Timoney, Daniel Prescott, Hamish D. C. Bain, Tasneem Bangi, Jerome McIntosh, Robin Egdell, R. Bruce Irwin, David Clark, Erik B. Schelbert, Gaetano Nucifora, Matthias Schmitt, Christopher A. Miller

**Affiliations:** 1 Wythenshawe Hospital, Manchester University NHS Foundation Trust, Southmoor Road, Manchester M23 9LT, UK; jakub.lagan@nhs.net (J.L.); christien.fortune@doctors.org.uk (C.F.); david.hutchings-2@manchester.ac.uk (D.H.); joshua.bradley@postgrad.manchester.ac.uk (J.B.); david.clark2@mft.nhs.uk (D.C.); gaetano.nucifora@mft.nhs.uk (G.N.); matthias.schmitt@mft.nhs.uk (M.S.); 2 Division of Cardiovascular Sciences, School of Medical Sciences, University of Manchester, Oxford Road, Manchester M13 9PL, UK; josephine.naish@manchester.ac.uk; 3 Stockport NHS Foundation Trust, Stepping Hill Hospital, Poplar Grove, Stockport SK2 7JE, UK; 03rtimoney@googlemail.com; 4 Bolton NHS Foundation Trust, Royal Bolton Hospital, Minerva Road, Bolton BL4 OJR, UK; djprescott92@gmail.com; 5 Wrightington, Wigan and Leigh NHS Foundation Trust, Royal Albert Edward Infirmary, Wigan Lane, Wigan WN1 2NN, UK; hdcbain@gmail.com; 6 Tameside and Glossop Integrated Care NHS Foundation Trust, Tameside Hospital, Fountain St., Ashton-under-Lyne OL6 9RW, UK; tasneem.bangi@doctors.org.uk (T.B.); jmcintosh@doctors.org.uk (J.M.); 7 Trafford General Hospital, Manchester University NHS Foundation Trust, Moorside Road, Manchester M41 5SL, UK; 8 East Cheshire NHS Trust, Macclesfield District General Hospital, Victoria Road, Macclesfield SK10 3BL, UK; robin.egdell@nhs.net; 9 Northern Care Alliance NHS Foundation Trust, Fairfield General Hospital, Rochdale Old Rd., Bury BL9 7TD, UK; Bruce.Irwin@pat.nhs.uk; 10 Department of Medicine, University of Pittsburgh School of Medicine, Pittsburgh, PA 15213, USA; schelberteb@upmc.edu; 11 UPMC Cardiovascular Magnetic Resonance Center, Heart and Vascular Institute, Pittsburgh, PA 15213, USA; 12 Wellcome Centre for Cell-Matrix Research, Manchester Academic Health Science Centre, University of Manchester, Oxford Road, Manchester M13 9PT, UK

**Keywords:** myocarditis, magnetic resonance imaging, parametric mapping

## Abstract

Cardiovascular magnetic resonance (CMR) is used to investigate suspected acute myocarditis, however most supporting data is retrospective and few studies have included parametric mapping. We aimed to investigate the utility of contemporary multiparametric CMR in a large prospective cohort of patients with suspected acute myocarditis, the impact of real-world variations in practice, the relationship between clinical characteristics and CMR findings and factors predicting outcome. 540 consecutive patients we recruited. The 113 patients diagnosed with myocarditis on CMR performed within 40 days of presentation were followed-up for 674 (504–915) days. 39 patients underwent follow-up CMR at 189 (166–209) days. CMR provided a positive diagnosis in 72% of patients, including myocarditis (40%) and myocardial infarction (11%). In multivariable analysis, male sex and shorter presentation-to-scan interval were associated with a diagnosis of myocarditis. Presentation with heart failure (HF) was associated with lower left ventricular ejection fraction (LVEF), higher LGE burden and higher extracellular volume fraction. Lower baseline LVEF predicted follow-up LV dysfunction. Multiparametric CMR has a high diagnostic yield in suspected acute myocarditis. CMR should be performed early and include parametric mapping. Patients presenting with HF and reduced LVEF require closer follow-up while those with normal CMR may not require it.

## 1. Introduction

Cardiovascular magnetic resonance (CMR) is increasingly used to investigate patients with suspected acute myocarditis [1]. However, the majority of data supporting the use of CMR for this indication is retrospective, and few studies have included contemporary parametric mapping techniques [2,3,4,5,6,7]. As a consequence, while CMR is included in guidelines and position statements regarding the investigation of suspected myocarditis, the Class of Recommendation varies and it is with Level of Evidence C, i.e., “consensus of opinion of the experts and or small studies, retrospective studies, registries” [1,8,9].

This study aimed to investigate the clinical utility of CMR in a large prospective cohort of patients with suspected acute myocarditis. The study also aimed to evaluate the impact that real-world variations in practice have on CMR findings, the relationship between clinical characteristics and CMR findings and factors that predict outcome.

## 2. Materials and Methods

### 2.1. Study Design and Participants

This was a prospective longitudinal cohort research study approved by the North West–Greater Manchester West Research Ethics Committee of the UK National Research Ethics Service. Written informed consent was obtained from all participants. The work was conducted according to the Helsinki Declaration.

Consecutive consenting adult patients undergoing clinical CMR for suspected acute myocarditis at Manchester University NHS Foundation Trust, UK, between 1 January 2015 and 31 December 2018, were prospectively recruited. The clinical suspicion of myocarditis was determined from the CMR referral information.

Patients undergoing CMR within 40 days of hospital presentation and without an alternative diagnosis were included in analyses investigating the relationship between baseline factors, CMR findings and outcome. A 40-day cut-off was chosen because this is in keeping with previous studies, and because the study aimed to investigate the impact of a real-world presentation-to-scan interval, which, in the authors’ experience, is up to around 6 weeks, even in a healthcare system relatively well served by CMR [10]. Patients diagnosed with acute myocarditis on baseline CMR and who underwent follow-up CMR were included in an additional analysis.

### 2.2. Study Procedures

Data were managed using Research Electronic Data Capture (REDCap) [11]. Acute presentation and baseline comorbidity data were determined from primary and secondary care medical records.

CMR was performed on 2 scanners (1.5 T Avanto and 3 T Skyra; Siemens, Munich, Germany) and included steady-state free precession cine imaging (standard long- and short-axis views), basal and mid LV short-axis T1 mapping (MOdified Look-Locker Inversion Recovery) acquired pre- and 15 min post-administration of gadolinium-based contrast agent (0.15–0.2 mmol/kg; gadoterate meglumine (Dotarem), Guerbet, France), T2 mapping (T2-prepared steady-state free precession) and late gadolinium enhancement (LGE) imaging.

### 2.3. CMR Analysis

CMR image analysis was performed using CVI42 (Circle Cardiovascular Imaging, Calgary, AB, Canada) according to current guidelines [12]. Mean relaxation times and their standard deviation are reported, the latter as an indication of heterogeneity, calculated after dividing regions of interest into 100 radii. Extracellular volume (ECV) was calculated as described previously [13]. Foci of non-ischaemic scar on LGE imaging were included in ECV measurements [14]. LGE was manually quantified from a LV short axis LGE stack [15]. CMR analysis was performed blinded to patient outcomes.

### 2.4. Study Outcomes

CMR diagnosis was made by the clinical reporting physician (Level 3 trained), independent of the research team. The primary endpoint for the outcome analysis was a composite of first hospitalisation for heart failure (HF) after CMR or all-cause mortality. First hospitalisation for HF was recorded from primary and secondary care medical records and determined independently by the clinical team responsible for the patient’s care. Mortality status was ascertained from primary and secondary care medical records.

### 2.5. Statistical Analysis

Data are summarised using mean and standard deviation or median and interquartile range (IQR), and were compared using t tests or non-parametric equivalents, as appropriate. χ^2^ tests were used to compare categorical variables. Logistic regression models (univariable and backward stepwise multivariable) were used to investigate the relationship between baseline clinical features and a diagnosis of myocarditis on CMR. Linear regression models (univariable and backward stepwise multivariable) were used to investigate the relationship between baseline factors and change in LV ejection fraction on follow-up CMR. The low numbers of patients that experienced the primary endpoint precluded meaningful outcome analysis, but exploratory univariable Cox regression analysis to evaluate the relationship between baseline factors and outcome was performed. Analyses were performed using SPSS (version 22, IBM, Armonk, NY, USA).

## 3. Results

### 3.1. Diagnostic Yield of CMR

**Figure 1:**
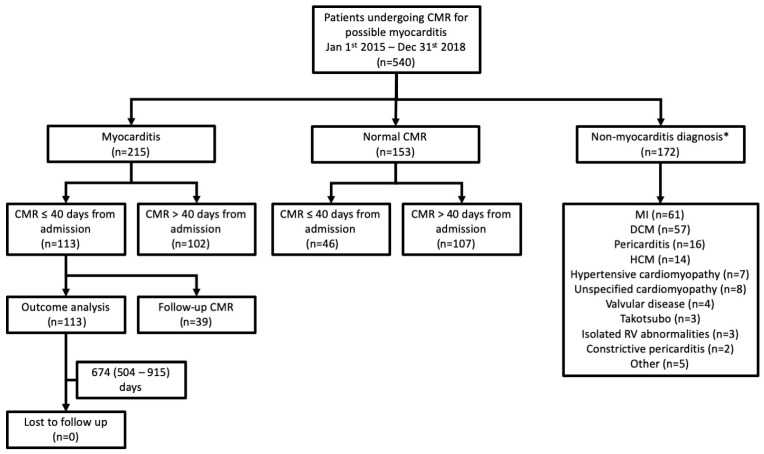
Derivation of the study cohort. CMR-cardiovascular magnetic resonance. * patients could have more than one diagnosis.

The cohort consisted of 540 patients; median age 47 years (IQR 33–60 years); 209 (39%) were female. Baseline characteristics are summarised in Table 1 and Figure 1. The most common presenting symptom was chest pain (354 patients; 66%). Palpitations or arrhythmia (68 patients; 13%) and symptoms of heart failure (66 patients; 12%) were less common. Viral prodrome was uncommon (50 patients, 9%).

CMR provided a positive diagnosis in 387 (72%) patients. The most common diagnoses were myocarditis (215 patients; 40%), myocardial infarction (MI; 61 patients; 11%) and dilated cardiomyopathy (57 patients; 11%). 

### 3.2. Relationship between Demographics, Laboratory Findings and Scan Timing, and CMR Diagnosis of Acute Myocarditis

After excluding patients with a positive ‘non-myocarditis’ diagnosis (172 patients), 159 patients underwent CMR within 40 days of presentation to hospital (Table 2; Figure 1). Of these 159 patients, 113 (71%) were diagnosed with myocarditis at CMR and 46 patients had normal CMR findings. 

Patients with a CMR in keeping with myocarditis were trending to be younger (40 (24–52) years vs. 44 (30–58) years; *p* = 0.074), were more often male (86 (76%) vs. 27 (24%); *p* < 0.001), had higher circulating inflammatory markers at presentation (e.g., c reactive protein 21 (4–67) mg/L vs. 3 (1–21) mg/L; *p* < 0.001) and more frequently had elevated circulating troponin levels (103 (96%) vs. 35 (76%); *p* < 0.001) than patients with normal CMR findings.

Patients diagnosed with myocarditis on CMR also had a shorter presentation–to–scan interval (15 (5–26) days vs. 26 (18–31) days; *p* < 0.001). Indeed, the interval between hospital presentation and CMR was negatively correlated with non-ischaemic LGE burden (r = −0.31, *p* < 0.001), myocardial T2 (1.5 T; r = −0.26, *p* = 0.020), T2 heterogeneity (r = −0.27, *p* = 0.002) and T1 heterogeneity (r = −0.24, *p* = 0.002). There was no correlation with ECV.

In multivariable analysis, only male gender (odds ratio (OR) 5.73, 95% confidence interval (95%CI) 2.42–13.54, *p* < 0.001) and shorter presentation–to–scan interval (OR 0.94, 95%CI 0.91–0.98, *p* = 0.003) were independently associated with a diagnosis of myocarditis on CMR.

### 3.3. Relationship between Clinical Presentation and CMR Measurements of Myocardial Injury

Presentation with chest pain was associated with higher LV ejection fraction (EF) (r = 0.43, *p* < 0.001), lower LGE burden (r = −0.17, *p* = 0.033) and lower ECV (r = −0.28, *p* < 0.001). Presentation with heart failure symptoms was associated with lower LV EF (r = −0.19, *p* = 0.015), higher LGE burden (r = 0.21, *p* = 0.008) and higher ECV (r = 0.22, *p* = 0.005). See Supplementary Table S1.

### 3.4. Factors Associated with LV Functional Recovery Following Acute Myocarditis

**Figure 2:**
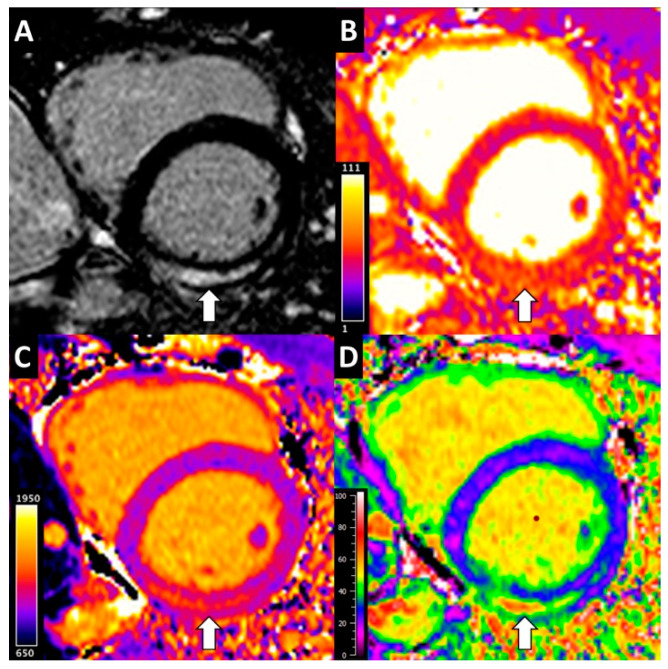
Multiparametric 1.5 T cardiac magnetic resonance in acute myocarditis. (**A**) Late gadolinium enhancement image showing focal enhancement in the inferolateral mid-wall (arrow). (**B**) T2 map. Inferolateral wall T2 elevated at 59 ms (arrow) compared to 47 ms in the anteroseptum. (**C**) Native T1 map. Inferolateral wall T1 elevated at 1286 ms (arrow) compared to 1009 ms in the anteroseptum. (**D**) Extracellular volume (ECV) map. Inferolateral wall ECV elevated at 53% (arrow) compared to 25% in the anteroseptum.

Thirty-nine patients found to have myocarditis on baseline CMR underwent follow-up CMR at a median 189 (166–209) days following hospital presentation. Left and right ventricular ejection fraction significantly increased and LV mass indexed for body surface area significantly reduced at follow-up compared to baseline (Table 3). Similarly, LGE burden, myocardial T1, T2 and ECV all significantly decreased. Representative parametric mapping is displayed in Figure 2.

In univariable analysis, higher baseline LV ejection fraction, lower baseline non-ischaemic LGE burden and lower baseline ECV were associated with higher LV ejection fraction at follow-up CMR (Table 4). In multivariable analysis, only baseline LV ejection fraction was independently associated with follow-up LV ejection fraction.

### 3.5. Factors Associated with Clinical Outcome Following Acute Myocarditis

The 113 patients diagnosed with myocarditis on CMR performed within 40 days of hospital presentation were followed up for a median of 674 (504–915) days. Four (3.5%) patients experienced the primary endpoint: two patients died (1 due to post-myocarditis dilated cardiomyopathy and 1 due to sepsis following non-cardiac surgery) and two patients were hospitalised for HF. The low numbers of patients that experienced the primary endpoint precluded meaningful outcome analysis. In exploratory univariable analysis, lower baseline LV ejection fraction (hazard ratio (HR) 0.87; 95% confidence interval (CI) 0.80–0.94) was associated with the primary endpoint. Presentation with chest pain was associated with a lower rate of the primary endpoint (HR 0.94; 95%CI 0.01–0.91). No patient with normal findings on CMR performed within 40 days of hospital presentation died or were hospitalised for HF during follow-up.

## 4. Discussion

This study is the largest prospective evaluation of the clinical utility of CMR in unselected patients referred with suspected acute myocarditis, and the first to include contemporary CMR techniques. 

The positive diagnostic yield in our study (72%) is higher than that in earlier studies (e.g., 52% in Biesbroek et al. [16]; 44% in Schumm et al. [17], likely in part reflecting the added diagnostic value of parametric mapping, which permits detection of myocardial oedema in the absence of necrosis and diffuse myocardial injury more effectively than conventional T2-weighted and LGE imaging [18]. Nevertheless, a substantial proportion of patients with suspected myocarditis have normal findings at CMR (28% in this study) [16,17]. Whilst the sensitivity of CMR appears to reduce over time from presentation (see below), there remains a significant number of patients for whom CMR is normal despite it being performed early. For example, Stensaeth et al. found 18% of patients had normal CMR findings despite the CMR being performed within 24 h of admission [19]. Coronary artery spasm, plaque disruption or thromboembolism with too little myonecrosis to be identified using the spatial resolution of CMR and ‘non-cardiac’ conditions have been proposed as possible mechanisms. Further investigation is required to understand the pathophysiology responsible for the clinical presentation in this group.

Being male and there being a shorter interval between hospital presentation and CMR were independently associated with a diagnosis of myocarditis on CMR. Population studies show myocarditis is more common in males [20]. The mechanism is unclear but preclinical studies have proven that males develop a greater myocardial inflammatory response to Coxsackievirus B infection [21]. Previous CMR studies in suspected myocarditis have shown non-ischaemic LGE to be more common in males [17,22].

Smaller studies have shown that the sensitivity of CMR for detecting myocarditis is higher when performed within 2 weeks of symptom onset compared to beyond 2 weeks, although these studies have generally not corrected for potential confounding factors such as sex [23,24]. Our study advances these earlier findings, demonstrating that shorter presentation–to–scan interval is independently associated with a CMR diagnosis of myocarditis. Indeed, shorter presentation–to–scan interval was associated with multiple metrics of myocardial injury severity, likely reflecting the time course of myocardial injury resolution [25].

This study is the largest to assess the relationship between clinical presentation and myocardial injury. Presentation with chest pain was associated with less myocardial injury and better LV function whereas HF symptoms were associated with greater myocardial injury (higher ECV, more non-ischaemic LGE) and lower LV function. These findings provide mechanistic insight into clinical myocarditis studies, which have shown that people presenting with chest pain have a better prognosis than those presenting with HF [26,27]. The relationship between clinical presentation and CMR findings in previous smaller CMR studies has been conflicting [17,28].

Early studies found endomyocardial biopsy proven-myocarditis to be associated with poor prognosis [29]. The outcome following myocarditis in our study (adverse event rate approximately 2% per year) is much more favourable and is in keeping with other contemporary studies of CMR-diagnosed myocarditis. Indeed, the low event rate precluded multivariable Cox regression analysis. The prognostic value of CMR parameters has varied across previous studies, although lower baseline LV ejection fraction and higher baseline LGE burden are factors that more commonly associate with adverse outcome [17,22,28]. In keeping with these findings, baseline LV ejection fraction was independently associated with follow-up LV ejection fraction in our study. Higher baseline LGE burden and ECV were associated with less LV recovery on univariable analysis, but the associations were no longer present after multivariable adjustment, albeit in the setting of limited statistical power. Further investigation is required.

Putting the findings of this study together with those of other studies, CMR for suspected myocarditis should be performed as early as possible following admission and include parametric mapping. Patients presenting with HF symptoms, demonstrating reduced LV ejection fraction and higher LGE burden require close clinical and imaging follow-up. Those with a normal scan may not require routine follow-up.

Our study provides prospective evidence from a large cohort to add to the already extensive, albeit largely retrospective, observational data to support the use of CMR for suspected acute myocarditis. A positive diagnostic yield of 3 in 4 patients is remarkable by any standard. Indeed, the strength of the observational data means that a higher level of evidence may preclude the field from achieving a higher Level of Evidence; a randomised trial comparing the utility of CMR with other imaging modalities for the diagnosis of, and prognosis following, acute myocarditis would be considered unethical. Endomyocardial biopsy is rarely performed and its own accuracy is limited, thus it is an inadequate comparator. Nevertheless, the consequence of not having randomised data is that CMR may remain as being recommended with only a “moderate level of consensus” and below that of other imaging modalities [8]. The advent of a therapy for myocarditis requiring a positive diagnosis for patient selection, and quantitative monitoring of myocardial response, may drive stronger recommendations. For example, entry into the ongoing trial of an IL-1 receptor antagonist in myocarditis (NCT03018834) requires “myocarditis proven by MRI”.

## 5. Conclusions

Contemporary multiparametric CMR has a high diagnostic yield in patients with suspected acute myocarditis. Being male and there being a shorter interval between hospital presentation and CMR are associated with a diagnosis of myocarditis on CMR. Presentation with chest pain is associated with less myocardial injury and better LV function than presentation with symptoms of HF. Clinical outcome following CMR-diagnosed myocarditis is favourable. Lower baseline LV ejection fraction predicted less recovery of LV function.

## 6. Limitations

Patients did not undergo endomyocardial biopsy, however it is rarely performed in most centres in patients with suspected myocarditis, and due to sampling error, transiency of myocardial injury and variation in histology interpretation, it is limited as a reference standard [30]. It is recognised that using myocardial injury may have lessened or resolved in the 40-day window between presentation and CMR, however this time frame was chosen because it is in keeping with real-world clinical practice, and understanding the impact that presentation–to–scan interval has on diagnostic yield was an important aim of the study. The survival analysis was limited by a low number of adverse events, as discussed. Single centre data may not generalise, but our findings align with prior literature.

## Figures and Tables

**Table 1:** Baseline characteristics.

Parameter	All Patients (*n* = 540)	Females (*n* = 209)	Males (*n* = 331)	*p* Value ^∆^
**Demographics**				
Age (Years)	47 (33–60)	52 (41–64)	44 (29–56)	<0.001
Gender (Female)	209 (39%)			
Presenting symptoms *				
Chest pain (n;%)	354 (66%)	147 (70%)	207 (63%)	0.077
Palpitations/arrhythmia (n;%)	68 (13%)	25 (12%)	43 (13%)	0.791
Heart failure (n;%)	66 (12%)	26 (12%)	40 (12%)	0.894
Viral prodrome (n;%)	50 (9%)	13 (6%)	37 (11%)	0.067
Systemic infection (n;%)	33 (6%)	9 (4%)	24 (7%)	0.198
Pre-/syncope (n;%)	22 (4%)	8 (4%)	14 (4%)	1.000
Generally unwell (n;%)	18 (3%)	6 (3%)	12 (4%)	0.807
GI symptoms (n;%)	17 (3%)	6 (3%)	11 (3%)	1.000
Cardiac arrest (n;%)	6 (1%)	2 (1%)	4 (1%)	1.000
Hypotension (n;%)	4 (1%)	1 (<1%)	3 (1%)	1.000
**CMR diagnosis ^†^**				
Myocarditis (n;%)	215 (40%)	55 (26%)	160 (48%)	< 0.001
Normal scan (n;%)	153 (28%)	90 (43%)	63 (19%)	<0.001
Myocardial infarction (n;%)	61 (11%)	26 (12%)	35 (11%)	0.577
Reversible Ischaemia (n;%)	2 (<1%)	1 (<1%)	1 (<1%)	1.000
HCM (n;%)	14 (3%)	8 (4%)	6 (2%)	0.171
DCM (n;%)	57 (11%)	13 (6%)	44 (13%)	0.009
Hypertensive cardiomyopathy (n;%)	7 (1%)	1 (<1%)	6 (2%)	0.257
Unspecified cardiomyopathy (n;%)	8 (1%)	4 (2%)	4 (1%)	0.717
Pericarditis (n;%)	16 (3%)	6 (3%)	10 (3%)	1.000
Constrictive pericarditis (n;%)	2 (<1%)	0 (0%)	2 (<1%)	0.525
Valvular disease (n;%)	4 (1%)	1 (<1%)	3 (1%)	1.000
Takotsubo (n;%)	3 (1%)	3 (1%)	0 (0%)	0.057
Isolated RV abnormalities (n;%)	3 (1%)	1 (<1%)	2 (<1%)	1.000
Vasculitis (n;%)	1 (<1%)	1 (<1%)	0 (0%)	1.000
Amyloid (n;%)	1 (<1%)	0 (0%)	1 (<1%)	1.000
Sarcoid (n;%)	1 (<1%)	0 (0%)	1 (<1%)	1.000

Data presented as mean ± standard deviation or median (interquartile range) depending on distribution. * Some patients had multiple symptoms; ^†^ Some patients had more than one diagnosis. ^∆^ females vs. males; CMR-Cardiovascular magnetic resonance; DCM-Dilated cardiomyopathy; GI-gastrointestinal; HCM-hypertrophic cardiomyopathy.

**Table 2:** Characteristics of patients undergoing CMR within 40 days of presentation (*n* = 159).

Parameter	Myocarditis (*n* = 113)	Normal (*n* = 46)	*p* Value
**Demographics**			
Age (years)	40 (24–52)	44 (30–58)	0.074
Gender (female)	27 (24%)	31 (67%)	<0.001
Presentation to scan interval (days)	15 (5–26)	26 (18–31)	<0.001
**Laboratory findings**			
WBC (×10^9^/L) *	9.3 (7.2–12.2)	8.1 (6.5–10.4)	0.044
CRP (mg/L) ^†^	21 (4–67)	3 (1–21)	<0.001
Abnormal troponin (n;%) ^∆^	103 (96%)	35 (76%)	<0.001
**CMR findings**			
LV EDV/BSA (mL/m^2^)	84 ± 16	79 ± 15	0.058
LV ESV/BSA (mL/m^2^)	33 ± 13	26 ± 8	<0.001
LV EF (%)	61 ± 9	67 ± 6	<0.001
LV mass/BSA (g/m^2^)	65 ± 13	53 ± 12	<0.001
RV EDV/BSA (mL/m^2^)	90 ± 17	82 ± 16	0.006
RV ESV/BSA (mL/m^2^)	40 ± 11	31 ± 9	<0.001
RV EF (%)	56 ± 7	62 ± 6	<0.001
LA area/BSA (cm^2^/m^2^)	12 ± 2	12 ± 2	0.823
RA area/BSA (cm^2^/m^2^)	12 ± 2	11 ± 2	0.059
LGE (g)	5.5 (2.9–10.7)	0.0 (0.0–0.0)	<0.001
LGE present (n;%)	109 (97%)	0 (0%)	<0.001
T2 mapping (ms) ^µ^			
1.5 T	50 (49–52)	49 (46–50)	0.055
3 T	41 (39–43)	40 (38–42)	0.434
SD	4.2 (3.5–5.2)	3.9 (3.1–4.8)	0.096
T1 mapping (ms)			
1.5 T	1044 (1018–1079)	1019 (1008–1052)	0.023
3 T	1244 (1221–1274)	1224 (1201–1264)	0.112
SD	56.4 (45.6–72.4)	46.9 (40.5–56.9)	<0.001
ECV (%) ^∂^	27.08 (25.4–30.3)	26.10 (24.26–27.87)	0.029

Data presented as mean ± standard deviation or median (interquartile range) depending on distribution. * *n* = 156; ^†^ *n* = 138; ^∆^ *n* = 152; ^µ^ *n* = 122; ^∂^ *n* = 155. BSA-Body surface area; CRP-C-reactive protein; ECV-extracellular volume; EF-Ejection fraction; EDV-End diastolic volume; ESV-End systolic volume; LA-Left atrium; RA-Right atrium; LGE-Late gadolinium enhancement; LV-Left ventricular; RV-Right ventricle; SD-Standard deviation; WBC-White blood cell. Other abbreviations as per Table 1.

**Table 3:** Characteristics of patients undergoing baseline and follow-up CMR (*n* = 39).

Parameter	Baseline	Follow-Up	*p* Value
**Demographics**			
Age (years)	33.6 (22.2–45.4)		
Presentation to scan interval (days)	5 (3–13)	189 (166–209)	
Gender (female)	7 (18%)		
**CMR findings**			
LV EDV/BSA (mL/m^2^)	88 ± 15	88 ± 14	0.691
LV ESV/BSA (mL/m^2^)	35 ± 11	33 ± 10	0.086
LV EF (%)	60 ± 8	63 ± 7	0.04
LV mass/BSA (g/m^2^)	68 ± 11	63 ± 10	0.002
RV EDV/BSA (mL/m^2^)	95 ± 13	96 ± 14	0.367
RV ESV/BSA (mL/m^2^)	44 ± 10	41 ± 9	0.045
RV EF (%)	55 ± 7	57 ± 6	0.023
LA area/BSA (cm^2^/m^2^)	12 ± 2	12 ± 2	0.806
RA area/BSA (cm^2^/m^2^)	12 ± 2	12 ± 2	0.705
LGE (g)	6.9 (4.0–16.6)	3.06 (1.8–6.5)	<0.001
T2 mapping (ms) *			
1.5 T	50 (49–52)	47 (46–49)	<0.001
3 T	40 (38–44)	37 (36–41)	0.046
SD	4.7 (3.9–5.7)	3.9 (3.3–4.6)	<0.001
T1 mapping (ms)			
1.5 T	1054 (1026–1089)	1015 (991–1030)	<0.001
3 T	1251 (1227–1423)	1224 (1167–1312)	0.028
SD	62.0 (48.1–77.5)	47.1 (39.1–56.7)	0.006
ECV (%)	27.13 (25.39–30.99)	25.87 (24.34–28.18)	<0.001

Data presented as mean ± standard deviation or median (interquartile range) depending on distribution. * *n* = 36 for baseline scan and *n* = 34 for follow up scan. Abbreviations as per Table 1 and Table 2.

**Table 4:** Factors associated with left ventricular recovery following acute myocarditis (*n* = 39).

Baseline Variables	B (SE)	*t* Value	*p* Value
**Univariate models**			
Age	0.12 ± 0.09	1.379	0.176
Gender (male)	1.38 ± 2.95	0.468	0.643
CRP (per 1 mg/L increase) *	−0.01 ± 0.014	−0.357	0.723
Troponin I (per 100 ng/L increase) ^†^	−0.02 ± 0.01	−1.234	0.226
T2 (per 1 ms increase) ^µ^	−0.50 ± 0.38	−1.309	0.201
T1 (per 1 ms increase) ^∂^	−0.01 ± 0.02	−0.591	0.559
ECV (per 1% increase)	−0.41 ± 0.23	−1.779	0.083
LGE (per 1 g increase)	−0.27 ± 0.10	−2.699	0.010
LV EF (per 1% increase)	0.50 ± 0.13	3.858	<0.001
**Multivariable model ^∆^**			
LV EF (per 1% increase)	050 ± 0.13	2.858	<0.001

* *n* = 37; ^†^ *n* = 36; ^µ^ *n* = 30, 1.5 T; ^∂^ *n* = 33, 1.5 T. ^∆^ Variables with a *p* value of less than 0.1 were included in the in the multivariable model (i.e., ECV, LGE, LV EF). Abbreviations as per Table 1, Table 2 and Table 3.

## Data Availability

The datasets generated and/or analysed during this study are available from the corresponding author on reasonable request.

## Supplementary Materials

The following are available online at https://www.mdpi.com/article/10.3390/diagnostics12010156/s1, Supplementary Table S1. Relationship between clinical presentation and cardiovascular magnetic resonance measurements.
