# Supplementary material for: The Diagnostic and Prognostic Utility of Contemporary Cardiac Magnetic Resonance in Suspected Acute Myocarditis
Source: Diagnostics (Basel). 2022 Jan 10;12(1):156. doi: 10.3390/diagnostics12010156 (PMC8774755; doi:10.3390/diagnostics12010156)
Supplement: Supplementary file 1 [file diagnostics-12-00156-s001.zip › diagnostics-1526176-supplementary.pdf]

The diagnostic and prognostic utility of contemporary CMR in suspected acute myocarditis

Supplementary material

Supplementary Table S1. Relationship between clinical presentation and cardiovascular magnetic resonance measurements.

Symptoms	LVEF (%)		LGE (g)		ECV (%)*	
	r	*p* value	r	*p* value	r	*p* value
**Chest pain**	0.43	<0.001	−0.17	0.033	−0.28	<0.001
**HF symptoms**	−0.19	0.015	0.21	0.008	0.22	0.005
**Pre-/Syncope**	−0.16	0.050	0.26	0.001	0.15	0.068
**Palpitations or arrhythmia**	−0.29	<0.001	0.03	0.678	0.09	0.264
**Troponin**†	0.09	0.257	0.18	0.023	0.09$^{\Delta}$	0.280

† n=152; * n=155; $^{\Delta}$ n=149. ECV-extracellular volume; HF-heart failure; LGE-Late enhancement imaging; LVEF-Left ventricular ejection fraction.
